# Coagulation status and determinants of possible aspirin resistance in patients with essential thrombocythemia

**DOI:** 10.3389/fmed.2022.1092281

**Published:** 2022-12-20

**Authors:** Erpeng Yang, Yan Lv, Ziqing Wang, Dehao Wang, Yumeng Li, Yan Sun, Yanyu Zhang, Jicong Niu, Zhuo Chen, Weiyi Liu, Xiaomei Hu

**Affiliations:** ^1^Department of Hematology, Xiyuan Hospital, China Academy of Chinese Medical Sciences, Beijing, China; ^2^Xiyuan Clinical Medical College, Beijing University of Traditional Medicine, Beijing, China

**Keywords:** essential thrombocythemia, aspirin resistance, clinical features, coagulation status, next-generation sequencing

## Abstract

**Objectives:**

The currently recommended aspirin regimen appears inadequate for thromboprophylaxis in essential thrombocythemia (ET). This study aimed not only to evaluate the curative effect of aspirin but also to explore the coagulation status and determinants of aspirin resistance (AR) of ET patients.

**Methods:**

A total of 80 ET patients who underwent coagulation tests, thromboelastography (TEG), and next-generation sequencing (NGS) were involved in the study. Patients were divided into the aspirin sensitivity (AS) group and AR group according to the arachidonic acid inhibition rate. Their clinical features and coagulation function were analyzed.

**Results:**

The incidence of AR was 53.75% (43/80) in 80 ET patients. Fbg was significantly higher in coagulation tests in AR patients compared with AS patients (*P* < 0.05), while the differences in other variables (D-D, PT, PTA, INR, APTT, TT, FDP, and AT-III) were not statistically significant (*P* > 0.05). Compared with AS patients, the *K* values, α angles, MA values, and CI values of TEG in AR patients were statistically smaller (*P* < 0.05), but there was no significant difference in *R* value between them (*P* > 0.05). Univariate and multivariate logistic regression analysis showed that age, irregular use of aspirin, smoking, dyslipidemia, and hypertension increased the risk of AR (*P* < 0.05). In the routine NGS, the driver gene and non-driver gene had no effect on AR in ET patients.

**Conclusion:**

Compared with AS patients, AR patients have enhanced platelet aggregation function, are in a relatively hypercoagulable state, and haveelevated fibrinogen function/levels, all of which cause a worse coagulation status. ET patients with increasing age, irregular use of aspirin, smoking, dyslipidemia, and hypertension are possibly at higher risk of AR. The routine NGS may not be helpful for the prediction of AR, therefore we recommend adding relevant drug-resistance genes to NGS.

## 1 Introduction

Essential thrombocythemia (ET) is a Philadelphia chromosome-negative myeloproliferative neoplasm (MPN) characterized by highly proliferative megakaryocytes in the bone marrow and markedly elevated platelet counts in peripheral blood (1). The arterial and venous thrombosis rate in MPN patients has been estimated as 3-fold and 10-fold increased, respectively, compared with the general population (2). Therefore, it is very important to prevent thrombosis in the treatment of these patients (3).

Low-dose aspirin (75–100 mg/day) is widely used to prevent thrombosis in patients with ET (4, 5). In recent years, studies have shown that patients’ responses to aspirin are different (6–8). In the clinic, even if some patients regularly take aspirin for a long time, thrombosis will still occur. This may be because aspirin has insufficient inhibitory effect on platelets. This phenomenon is called aspirin resistance (AR), while the opposite is called aspirin sensitivity (AS) (9, 10). The purpose of this study was not only to evaluate the curative effect of aspirin in ET patients, but also to explore the coagulation status and determinants of AR.

## 2 Materials and methods

### 2.1 Patients

We collected data from 80 ET patients in the Xiyuan Hospital from June 2019 to December 2021. All patients were diagnosed according to World Health Organization (WHO) diagnostic criteria (11) and underwent the next-generation sequencing (NGS), coagulation test, and thromboelastography (TEG). All patients were over 18 years old and had been taking aspirin (100 mg once a day) for at least 1 month. Patients could not take other drugs that affect coagulation function within one month before being examined. This study was approved by the medical ethics committee of the hospital (Reference number: 2019XLA024-3) and by the Chinese Clinical Trial Registry (Registry number: ChiCTR2200057736).

### 2.2 Clinical and laboratory data

Laboratory data included sex, age, aspirin use, cardiovascular risk factors (smoking, dyslipidemia, hypertension, and diabetes), history of thrombosis, presence or absence of splenomegaly, driver gene types (*JAK2*, *CALR*, *MPL*, and Triple negative), presence or absence of non-driver genes, routine blood test (WBC, HGB, PLT, NLR, and PLR), coagulation test (Fbg, D-D, PT, PTA, INR, APTT, TT, FDP, and AT-III), and TEG (R, K, α angle, MA, and CI). Referring to the relevant evaluation criteria (12–16), AR is defined as the inhibition rate of arachidonic acid (AA) <50%, and AS is defined as the inhibition rate of AA ≥50%.

### 2.3 Statistical analysis

SPSS 26.0 statistical software was used for analysis. The measurement data conforming to the normal distribution adopted the mean ± standard deviation (*x* ± *s*) and two-sample *t*-tests. If the measurement data did not conform to the normal distribution, *M* (P25 and P75) was used to express it, and the rank sum test was adopted. The risk factors of AR were analyzed by univariate and multivariate logistic regression. The enumeration data were statistically analyzed with the Chi-squared test. *P* < 0.05 meant statistically significant.

## 3 Results

### 3.1 The incidence of AR in ET patients

Among the 80 ET patients, 43 (53.75%) developed AR, with an average AA inhibition rate of 25.91%. There were 37 patients with AS, and the average inhibition rate of AA was 65.05%.

### 3.2 Comparison of clinical characteristics between AR and AS patients

Compared with AS patients, AR patients were significantly older, took aspirin irregularly, and had smoking, dyslipidemia, and hypertension (*P* < 0.05). There was no significant difference (*P* > 0.05) between the two groups in gender, thrombosis history, splenomegaly, driver gene type, white blood cell count (WBC), hemoglobin (HGB), platelet count (PLT), neutrophil-lymphocyte ratio (NLR), and platelet-lymphocyte ratio (PLR), as shown in Table 1.

**TABLE 1:** Comparison of clinical characteristics between AR and AS patients.

Variable	AR patients *n* = 43	AS patients *n* = 37	*P*
Sex			0.215
Male, *n* (%)	15 (34.88)	18 (48.65)	
Female, *n* (%)	28 (65.12)	19 (51.35)	
Age at enrollment (*x* ± *s* years)	55.26 ± 11.21	46.08 ± 14.04	0.002
Irregular use of aspirin	20 (46.51)	8 (21.62)	0.021
Cardiovascular risk factors	31 (72.09)	10 (27.03)	0.000
Smoking	9 (20.93)	2 (5.41)	0.046
Dyslipidemia	19 (44.19)	5 (13.51)	0.003
Hypertension	16 (37.21)	3 (8.11)	0.002
Diabetes	1 (2.33)	0	0.354
Thrombosis history	8 (18.60)	6 (16.22)	0.972
Splenomegaly	13 (30.23)	14 (37.84)	0.476
**Driver gene**			
*JAK2*+	26 (60.47)	24 (64.86)	0.687
*CALR*+	10 (23.26)	9 (24.32)	0.911
*MPL*+	1 (2.33)	0	0.354
Triple negative	6 (13.95)	4 (10.81)	0.674
**Routine blood test**			
WBC (×109/L)	7.70 (5.94, 9.85)	6.83 (5.29, 9.78)	0.559
HGB (g/L)	140.42 ± 18.89	146.27 ± 18.78	0.170
PLT (×109/L)	593.00 (488.00, 723.00)	599.00 (498.50, 708.50)	0.919
NLR	2.60 (1.69, 3.80)	2.70 (2.06, 3.91)	0.493
PLR	376.44 (278.99, 446.09)	377.89 (277.57, 471.85)	0.717

WBC, white blood cell; HGB, hemoglobin; NLR, neutrophil-lymphocyte ratio; PLR, platelet-lymphocyte ratio.

### 3.3 Comparison of coagulation status between AR patients and AS patients

In the coagulation test, the fibrinogen (Fbg) of 5% (4/80, 3 cases of AR and 1 case of AS) ET patients exceeded the normal upper limit, while other indexes (D-D, PT, PTA, INR, APTT, TT, FDP, and AT-III) did not. On TEG, 7.5% (6/80, 5 cases of AR and 1 case of AS) of patients had enhanced coagulation factor function (*R* < 4 min), 30.0% (24/80, 18 cases of AR and 6 cases of AS) of patients had increased fibrinogen function/level (*K* < 1 min or α angle >72°), 45.0% (36/80, 24 cases of AR and 12 cases of AS) of patients had enhanced platelet aggregation function (MA >70 mm), and 26.3% (21/80, 17 cases of AR and 4 cases of AS) of patients were in hypercoagulable state (CI >3).

Specifically, in the coagulation test, the Fbg of AR patients was significantly higher than that of AS patients (*P* < 0.05), while the differences in other variables (D-D, PT, PTA, INR, APTT, TT, FDP, and AT-III) were not statistically significant (*P* > 0.05). Compared with AS patients, the *K* values, α angles, MA values, and CI values of TEG in AR patients were lower (*P* < 0.05). But the difference of *R* values was not statistically significant (*P* > 0.05). See Table 2 for greater detail.

**TABLE 2:** Comparison of coagulation status between AR patients and AS patients.

Variable	AR patients (*n* = 43)	AS patients (*n* = 37)	*P*
**Coagulation test**			
D-D (mg/L)	0.260 (0.170, 0.380)	0.340 (0.160, 0.405)	0.727
PT (s)	11.800 (11.100, 12.100)	11.900 (11.400, 12.550)	0.115
PTA (%)	98.600 (95.400, 105.700)	97.500 (92.000, 103.250)	0.191
INR	1.030 (0.960, 1.050)	1.040 (0.990, 1.080)	0.176
APTT (s)	30.000 (27.800, 31.400)	30.800 (29.100, 32.750)	0.131
TT (s)	18.430 ± 1.422	18.787 ± 1.328	0.253
Fbg (g/L)	2.710 (2.310, 3.220)	2.430 (2.060, 2.745)	0.006
FDP (μg/ml)	2.000 (2.000, 2.000)	2.000 (1.050, 2.000)	0.072
AT-III (%)	98.630 ± 8.866	96.058 ± 10.520	0.239
**TEG**			
R (min)	5.200 (4.600, 6.700)	5.600 (5.150, 6.650)	0.123
K (min)	1.300 (1.000, 1.600)	1.600 (1.300, 1.850)	0.008
α angle (°)	70.093 ± 6.665	66.603 ± 5.153	0.012
MA (min)	71.412 ± 6.170	67.105 ± 5.220	0.001
CI	2.174 ± 2.172	1.119 ± 1.478	0.015

D-D, D-dimer; PT, prothrombin time; PTA, prothrombin time activity; INR, international normalized ratio; APTT, activated partial thromboplastin time; TT, thrombin time; Fbg, fibrinogen; FDP, fibrin degradation products; AT-III, antithrombin III; R, reaction time; K, clot kinetics; MA, maximum amplitude; CI, coagulation index.

### 3.4 Risk factors for AR: Univariate logistic regression analysis

Aspirin resistance was used as the dependent variable, as shown in Table 3, and 19 influencing factors were analyzed by univariate logistic regression. The results showed that patients with older age (OR = 1.059, 95% CI 1.019–1.101, *P* = 0.003), irregular aspirin use (OR = 3.152, 95% CI 1.176–8.447, *P* = 0.022), dyslipidemia (OR = 5.067, 95% CI 1.656–15.502, *P* = 0.004), and hypertension (OR = 6.716, 95% CI 1.772–25.460, *P* = 0.005) had a higher risk of AR.

**TABLE 3:** Univariate logistic regression analysis of AR.

Variable	Group	*B*	Standard error	Wald	*P*	OR	95% CI
Sex	Female	0.570	0.459	1.543	0.214	1.768	0.719–4.347
	Male*						
Age at enrollment		0.058	0.020	8.612	0.003	1.059	1.019–1.101
Use of aspirin	Irregular	1.148	0.503	5.211	0.022	3.152	1.176–8.447
	Regular*						
Smoking	Yes	1.533	0.818	3.513	0.061	4.632	0.932–23.018
	No*						
Dyslipidemia	Yes	1.623	0.571	8.088	0.004	5.067	1.656–15.502
	No*						
Hypertension	Yes	1.905	0.680	7.846	0.005	6.716	1.772–25.460
	No*						
Diabetes	Yes	21.076	40,192.969	0.000	1.000	1,423,156,409.178	N/A
	No*						
Thrombosis history	Yes	0.166	0.594	0.078	0.779	1.181	0.369–3.781
	No*						
Splenomegaly	Yes	-0.340	0.475	0.513	0.474	0.712	0.281–1.804
	No*						
*JAK2*+	Yes	-0.188	0.465	0.164	0.685	0.828	0.333–2.059
	No*						
*CALR*+	Yes	-0.059	0.526	0.013	0.911	0.943	0.336–2.645
	No*						
*MPL*+	Yes	21.076	40,192.969	0.000	1.000	1,423,156,409.178	N/A
	No*						
Triple negative	Yes	0.291	0.688	0.179	0.672	1.338	0.347–5.157
	No*						
Non-driving gene	Yes	-0.345	0.467	0.547	0.460	0.708	0.284–1.768
	No*						
WBC		-0.046	0.058	0.631	0.427	0.955	0.853–1.070
HGB		-0.017	0.012	1.878	0.171	0.983	0.960–1.007
PLT		0.000	0.001	0.042	0.838	1.000	0.998–1.002
NLR		-0.134	0.141	0.902	0.342	0.875	0.663–1.153
PLR		-0.001	0.001	0.900	0.343	0.999	0.997–1.001

*Control group. N/A, not applicable.

### 3.5 Risk factors for AR: Multivariate logistic regression analysis

Variables with *P* < 0.10 in the univariate logistic regression analysis were included in the multivariate logistic regression analysis. A total of five variables were eligible (age, irregular aspirin use, smoking, dyslipidemia, and hypertension). The results showed that patients who took aspirin irregularly (OR = 7.158 95% CI 1.973–25.964, *P* = 0.003), smoked (OR = 8.830, 95% CI 1.422–54.833, *P* = 0.019), had dyslipidemia (OR = 4.949, 95% CI 1.121–21.850, *P* = 0.035), and had hypertension (OR = 5.629, 95% CI 1.186–26.710, *P* = 0.030) had a higher risk of AR, as shown in Table 4.

**TABLE 4:** Multivariate logistic regression analysis of the influencing factors of AR.

Variable	Group	*B*	Standard error	Wald	*P*	OR	95% CI
Age at enrollment		0.040	0.027	2.265	0.132	1.041	0.988–1.097
Use of aspirin	Irregular	1.968	0.657	8.963	0.003	7.158	1.973–25.964
	Regular*						
Smoking	Yes	2.178	0.932	5.465	0.019	8.830	1.422–54.833
	No*						
Dyslipidemia	Yes	1.599	0.758	4.454	0.035	4.949	1.121–21.850
	No*						
Hypertension	Yes	1.728	0.794	4.731	0.030	5.629	1.186–26.710
	No*						

*Control group.

**FIGURE 1:**
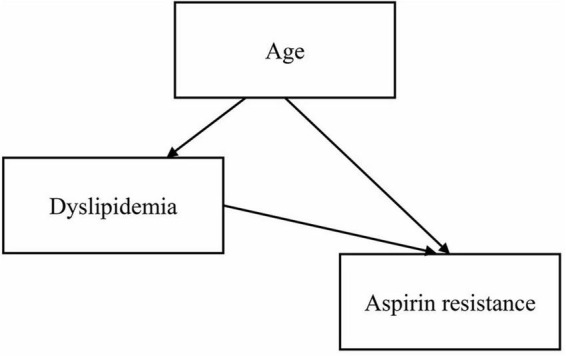
The directed acyclic graph among age, dyslipidemia, and aspirin resistance.

Age was statistically significant in the univariate logistic regression analysis, but not in the multivariate logistic regression analysis. The main reason could be the presence of intermediate or confounding variables. We used the two-factor model approach to explore the reasons for the inconsistent results. We established several regression models, each with the dependent variable “aspirin resistance,” the independent variable “age” and one other independent variable. Only in the two-factor regression model of “age + dyslipidemia,” age was found to be statistically insignificant, so “dyslipidemia” was considered to be an interfering factor affecting age. Studies have shown that age is a risk factor for dyslipidemia (17, 18), and a directed acyclic graph (DAG) showed that dyslipidemia is an intermediate variable of age (Figure 1). After excluding dyslipidemia, the other four variables (age, irregular aspirin use, smoking, and hypertension) were analyzed by multivariate logistic regression. The results showed that the older the patients were, the higher the risk of AR was (OR = 1.067, 95% CI 1.017–1.119, *P* = 0.008). The Chi-square test was used to further analyze the relationship between different age segments and AR, and there were significant differences (χ^2^ = 11.410, *P* = 0.022). It could be seen that the AR resistance rate gradually increased with age increase, especially after the age of 40, the proportion of AR patients was significantly higher than that of AS patients (as shown in Table 5).

**TABLE 5:** The Chi-square test analysis of the relationship between different age segments and AR.

Variable	Group	Age (%)					*n*	χ^2^	*P*
		**20–30 years**	**30–40 years**	**40–50 years**	**50–60 years**	**>60 years**			
AR	Yes	1 (14.29)	4 (28.57)	7 (53.85)	16 (66.67)	15 (68.18)	43 (53.75)	11.410	0.022*
	No	6 (85.71)	10 (71.43)	6 (46.15)	8 (33.33)	7 (31.82)	37 (46.25)		
*n*		7	14	13	24	22	80		

*p < 0.05.

### 3.6 Comparison of driver genes mutational load and non-driver genes types between AR and AS patients based on NGS

Table 1 demonstrated that there was no significant difference in the type of driver genes between AR and AS patients. As shown in Table 6, our further analysis showed that there was no significant difference in driver gene mutational load and non-driver gene types between AR and AS patients (*P* > 0.05).

**TABLE 6:** Comparison of driver genes mutational load and non-driver genes types between AR and AS patients.

Variable	AR patients (*n* = 43)	AS patients (*n* = 37)	*P*
**Driver gene mutational load**			
*JAK2* mutational load (%)	20.860 (14.570, 42.238)	20.485 (11.050, 40.573)	0.801
*CALR* mutational load (%)	24.864 ± 12.812	32.369 ± 13.487	0.231
*MPL* mutational load (%)	N/A	N/A	N/A
Non-driver gene type	14 (32.56)	15 (40.54)	0.462
DNA methylation (*TET2, DNMT3A, IDH1, IDH2*)	8 (18.60)	8 (21.62)	0.738
Histone modification (*ASXL1, EZH2, KMT2D, KMT2B*)	4 (9.30)	3 (8.11)	0.851
mRNA splicing (*SF3B1, SRSF2, U2AF1, ZRSR2*)	0	2 (5.41)	0.125
Signaling pathways (*LNK/SH2B3, CBL, NRAS/KRAS, PTPN11, ATG2B, ABCB1, NF1*)	3 (6.98)	1 (2.70)	0.385
Transcription factor (*RUNX1, NFE2, PPM1D, TP53, BCOR, WT1, ETV6*)	2 (4.65)	3 (8.11)	0.527

N/A, not applicable.

## 4 Discussion

Thrombosis prevention is an important therapeutic goal of ET. Aspirin is widely used for the primary and secondary prevention of thrombosis in ET patients. A study showed that the widely promoted low-dose (100 mg mg/day) aspirin regimen could not effectively reduce platelet activation (19). Therefore, exploring coagulation status and determinants of AR has important clinical significance for thromboprophylaxis of ET.

Thrombosis occurs as a result of a combination of changes in the vascular endothelial cells, platelets, coagulation, fibrinolytic system, and blood rheology. Studies have shown that all these factors have changed to varying degrees before thrombosis (3, 20, 21). TEG can dynamically monitor the process of coagulation, and it can help identify the prethrombotic state of patients in combination with a coagulation test (22–24).

In the study, the incidence of AR was 53.75% in 80 patients. Our study found that 45.0% of ET patients had enhanced platelet aggregation (MA >70 mm), and 26.3% had hypercoagulability (CI >3). Specifically, the incidence of enhanced platelet aggregation was higher in AR patients than in AS patients (55.81 > 32.43%, *P* < 0.05), and the incidence of hypercoagulable state was also higher in AR patients than in AS patients (39.53 > 10.81%, *P* < 0.05). This means that even in AS patients, 1/3 of them still have enhanced platelet aggregation function, and 1/10 of them are in a hypercoagulable state. This indicates that the current antiplatelet treatment scheme is really inadequate and needs to be improved. And compared to AS patients, AR patients had significantly higher fibrinogen values, significantly lower *K* values, and significantly larger alpha angles (*p* < 0.05). Therefore, it is considered that hypercoagulability is not only related to the enhancement of platelet aggregation function, but also related to the function/level of fibrinogen. The *R* value can reflect the activity of coagulation factors. In this study, 7.5% of patients had *R* values below normal, which means that there is no general abnormality of coagulation factors in ET patients. And there was no difference in *R* values between AR patients and AS patients, which implies that aspirin has little effect on coagulation factors.

Aspirin can prevent the production of thromboxane A2 (TXA2) in platelets by irreversibly acetylating a serine residue at position 529 of the cyclooxygenase-1 (COX-1) isoform (25). Aging affects AR probably associated with some reduction in the first-pass metabolism and bioavailability of aspirin, which is due to decreased liver mass and perfusion (26, 27). Taking aspirin irregularly will weaken its efficacy, which is an important factor of AR (25). In smokers, the biosynthesis of TXA2 is increased. Their serum C-reactive protein (CRP) also increases, while in the inflammatory state, the risk of AR increases (28, 29). Hypercholesterolemia and hypertension can lead to overexpression of COX-1, which enhances AR (30–32). After univariate and multivariate logistic regression analysis, the risk of AR was higher in ET patients with increasing age, irregular aspirin use, smoking, dyslipidemia, and hypertension. It suggests that ET patients should take aspirin regularly, quit smoking, and control blood lipid and blood pressure. However, there are too few diabetic patients among these patients to accurately judge the relationship between diabetes and AR, which is still worthy of attention.

Polymorphisms in some genes (COX-1, COX-2, GPIba, etc.) are strongly associated with AR, but it is still unknown whether the ET’s driver and non-driver genes affect it (33, 34). Unfortunately, our study showed that the driver and non-driver genes do not assist in predicting AR in ET patients. This reminds us of the need to add relevant drug-resistance genes to the routine NGS.

## 5 Conclusion

Compared with AS patients, AR patients have enhanced platelet aggregation function, are in a relatively hypercoagulable state, and have elevated fibrinogen function/levels, all of which cause a worse coagulation status. ET patients with increasing age, irregular aspirin use, smoking, dyslipidemia, and hypertension may have a higher risk of AR. The routine NGS may not be helpful for the prediction of AR, therefore we recommend adding relevant drug-resistance genes to NGS.

## Data availability statement

The original contributions presented in this study are included in the article/supplementary material, further inquiries can be directed to the corresponding authors.

## Ethics statement

The studies involving human participants were reviewed and approved by the Medical Ethics Committee of China Academy of Chinese Medical Sciences Xiyuan Hospital. The patients/participants provided their written informed consent to participate in this study. Written informed consent was obtained from the individual(s) for the publication of any potentially identifiable images or data included in this article.

## Author contributions

EY and XH designed the study. ZW collected the data. YuL, DW, YS, and YZ performed the analysis. JN and ZC normalized the pictures. EY, YaL, and WL wrote the original draft. All authors contributed to the article and approved the submitted version.
